# Pass Him Around

**Published:** 1867

**Authors:** 


*Pass him Around.*—A correspondent sends us a doubly distilled quack advertising sheet, illuminated (this is not a Hibernicism—Mrs. Browning writes of "raying out blackness") with portentous and hideous cuts of decayed lungs, hare-lips *et cetera*, connected by letter text wherein most wonderful cures are announced (and promised), and winding up with the inevitable "Manhood" and Spermatorrhœa dodge. The paper purports to be edited at Chicago and issued periodically by one A. S. HUDSON, M.D., who claims further consideration as having formerly been connected with Rush Medical College. Whatever may have been his former associations, it is perhaps sufficient to say that by his present course he has forfeited any professional respect or position. A man cannot meddle with pitch without being defiled, and Dr. H. has caught the double *Tar-tar*.

During the sessions of 1859–60, Dr. H. attempted a course of lectures in Rush College on Physiology and Pathology. Students in attendance upon that occasion will recollect that his course was brief enough, but scarcely glorious. We believe he retired before the close of the term, scarcely standing upon the order of his going, leaving the class to exclaim with regard to his lectures:

"When we think how quickly they were done for,
We wonder what they were begun for!"

However, as Dr. H. is skilled in the use of chalk, it is to be supposed he has chalked out a course for himself from which he cannot be dissuaded. In regard to it we can scarcely say with Prince Hal:

"We could have better spared a better man!"

We mourn the defection, but not as those without hope—of being able to endure it.

This *Hudson* is passing through the *Hellgate* of quackery to the *Atlantic* of professional scorn. We pity any man who thinks he can sacrifice even a medocre professional position, albeit with limited rewards, for all the wealth which quackery however successful, can offer.

